# A Multidrug Approach to Modulate the Mitochondrial Metabolism Impairment and Relative Oxidative Stress in Fanconi Anemia Complementation Group A

**DOI:** 10.3390/metabo12010006

**Published:** 2021-12-21

**Authors:** Enrico Cappelli, Nadia Bertola, Silvia Bruno, Paolo Degan, Stefano Regis, Fabio Corsolini, Barbara Banelli, Carlo Dufour, Silvia Ravera

**Affiliations:** 1 Hematology Unit, IRCCS Istituto Giannina Gaslini, Via Gerolamo Gaslini 5, 16148 Genova, Italy; enrico.cappelli@brogno.it (E.C.); carlodufour@gaslini.org (C.D.); 2 Department of Experimental Medicine, University of Genoa, Via De Toni 14, 16132 Genova, Italy; nadia.bertola@edu.unige.it (N.B.); silvia.bruno@unige.it (S.B.); 3 U.O. Mutagenesis, IRCCS AOU San Martino—IST (Istituto Nazionale per la Ricerca sul Cancro), Largo Rosanna Benzi 10, 16132 Genova, Italy; paolo.degan@virgilio.it; 4 Laboratory of Clinical and Experimental Immunology, IRCCS Istituto Giannina Gaslini, Via Gerolamo Gaslini 5, 16148 Genoa, Italy; stefanoregis@gaslini.org; 5 Centro di Diagnostica Genetica e Biochimica delle Malattie Metaboliche, IRCCS Istituto Giannina Gaslini, Via Gerolamo Gaslini 5, 16148 Genova, Italy; fabiocorsolini@gaslini.org; 6 Laboratory of Tumor Epigenetics, IRCCS AOU San Martino—IST, Largo Rosanna Benzi 10, 16132 Genova, Italy; banellib.epigenetics@gmail.com

**Keywords:** fanconi anemia, fatty acid synthesis, lipid accumulation, mitochondrial metabolism, oxidative stress, quercetin, rapamycin

## Abstract

Fanconi Anemia (FA) is a rare recessive genetic disorder characterized by aplastic anemia due to a defective DNA repair system. In addition, dysfunctional energy metabolism, lipid droplets accumulation, and unbalanced oxidative stress are involved in FA pathogenesis. Thus, to modulate the altered metabolism, Fanc-A lymphoblast cell lines were treated with quercetin, a flavonoid compound, C75 (4-Methylene-2-octyl-5-oxotetrahydrofuran-3-carboxylic acid), a fatty acid synthesis inhibitor, and rapamycin, an mTOR inhibitor, alone or in combination. As a control, isogenic FA cell lines corrected with the functional Fanc-A gene were used. Results showed that: (i) quercetin recovered the energy metabolism efficiency, reducing oxidative stress; (ii) C75 caused the lipid accumulation decrement and a slight oxidative stress reduction, without improving the energy metabolism; (iii) rapamycin reduced the aerobic metabolism and the oxidative stress, without increasing the energy status. In addition, all molecules reduce the accumulation of DNA double-strand breaks. Two-by-two combinations of the three drugs showed an additive effect compared with the action of the single molecule. Specifically, the quercetin/C75 combination appeared the most efficient in the mitochondrial and lipid metabolism improvement and in oxidative stress production reduction, while the quercetin/rapamycin combination seemed the most efficient in the DNA breaks decrement. Thus, data reported herein suggest that FA is a complex and multifactorial disease, and a multidrug strategy is necessary to correct the metabolic alterations.

## 1. Introduction

Fanconi Anemia (FA) is a rare autosomal or X-linked recessive disease [1], characterized by bone marrow failure and aplastic anemia, which represent the primary causes of death [2]. To date, 23 genes are involved in FA [3], which codify for proteins involved in a complex enlisted in response to genotoxic insults [4,5]. Thus, their pathogenic variants induce a defect in the DNA repair mechanisms and the cell cycle arrest in the G2 phase [6]. Fanc-A is the most frequently mutated FA gene, representing about two-thirds of the cases [7,8]. Its mutation spectrum is heterogeneous and seems to be correlated with the severity of the clinical phenotype [9].

Although the dysfunctional DNA repair was considered the main molecular hallmark of FA [5], other molecular mechanisms play a pivotal role in the deranged functioning of FA cells, such as energy metabolism alterations and the increment of oxidative stress production [10,11,12,13,14,15,16,17]. In particular, FA cells show a dysfunctional electron transport between the respiratory Complexes I and III that causes a shift from aerobic to anaerobic metabolism [13,14]. The altered oxidative phosphorylation (OxPhos) causes an increment of oxidative stress production [16,18], which is not counteracted by the endogenous antioxidant defenses [19]. Moreover, the biochemical alterations are associated with a defect in mitochondrial morphology [20,21]. Due to dysfunctional mitochondrial metabolism, FA cells accumulate acetyl-CoA, which leads to lipid droplets formation [22], causing a further defect in mitochondrial function [23]. Notably, the metabolic impairments in FA cells are correlated with insulin resistance (IR) and other clinical effects as obesity, and dyslipidemia, frequently observed in FA patients [24,25]. Interestingly, all these metabolic alterations are visible only in normoxic conditions, while in the bone marrow niche, a hypoxic environment, the mitochondrial defect is not detectable [26].

Hence, since mitochondria and the relative metabolism seem to play a pivotal role in FA, in this work, we treated FA cells with different drugs acting on these metabolic pathways, alone or in combination, to evaluate the interconnection between mitochondrial function, oxidative stress, and lipid metabolism. The results highlight the profound metabolic alterations in FA cells and suggest that, despite the metabolic interconnection, it is not sufficient to act on a single metabolic defect to enhance the whole metabolism.

## 2. Results

The experiments reported in this manuscript were done in the presence of the following molecules: (i) quercetin, an antioxidant molecule, (ii) C75, (4-Methylene-2-octyl-5-oxotetrahydrofuran-3-carboxylic acid) an inhibitor of fatty acid synthesis, and (iii) rapamycin, a modulator of pathways involved in mitochondrial metabolism.

### 2.1. Quercetin, C75, Rapamycin, and Their Combinations Slow Downs Cellular Growth and Limit the Cell Death

**Figure 1:**
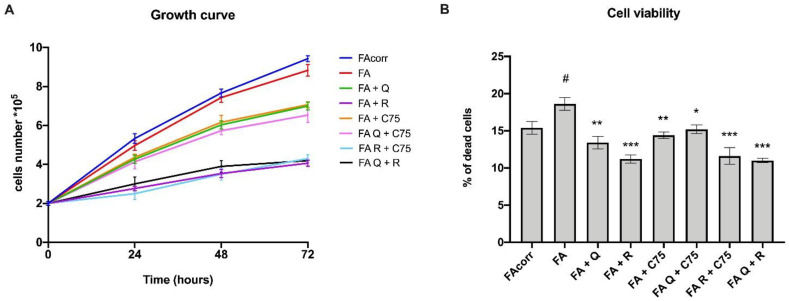
Reduced cell growth and dead in FA cells treated with quercetin, rapamycin, C75, and their combinations. (**A**) Cell growth curves. (**B**) Percentage of dead cells at 48 h. (Q = quercetin alone; R = rapamycin alone; C75 = C75 alone; Q + C75 = quercetin/C75 combination; R + C75 = rapamycin/C75 combination; Q + R = quercetin/rapamycin combination). Each graph represents four independent experiments and data are expressed as mean ± S.D. For FA and FA-corr cells, data are representative of four different Fanc-A lymphoblast cell lines. Statistical analysis was performed via one-way ANOVA followed by Tukey multiple comparison test. # indicates a *p* < 0.05 between FA cells and FA-corr cell lines; *, **, or *** indicate a *p* < 0.05, 0.01, or 0.001, respectively, between untreated and treated FA cells.

Despite the genetic defect, FA lymphoblastoid cell lines display a viability rate similar to their corrected counterparts, as reported in Figure 1A. In contrast, all drug treatments caused a slowdown in cell growth. Specifically, rapamycin and its combinations with quercetin or C75 show the lowest growth rate while quercetin, C75, and their combination induce a milder slow down (Figure 1A). However, despite the slower cell growth, treatments do not induce more cell death than untreated FA cells but significantly reduce it (Figure 1B). Thus, the data suggest that the proposed drugs are not detrimental to the FA cells viability.

### 2.2. Quercetin, C75, Rapamycin, and Their Combinations Modulate the Mitochondrial Aerobic Metabolism in FA Cells

FA cells are characterized by a dysfunctional mitochondrial aerobic metabolism due to a defect in the electron transport chain, which causes inefficient energy production, an increment in oxidative stress, and lipid droplets accumulation [13,14,15,16,17]. Therefore, as an attempt to modulate energy metabolism, FA cells were treated with quercetin, C75, rapamycin, and their combinations. 

Quercetin treatment caused contemporary the recovery of the electron transport between Complexes I and III, which appeared sharply lower in the untreated FA cells compared to the FA-corr (Figure 1A), and the reduction of oxygen consumption rate (OCR), mitochondrial membrane potential (MMP), and ATP synthesis compared to the untreated sample (Figure 1B–D). Interestingly, despite the reduction in mitochondrial activity, the efficiency of residual OxPhos increased similarly to that of FA-corr cells, as indicated by the P/O value (Figure 1E). In other words, quercetin slowed down aerobic metabolism, increased both electron transport and energy production efficiency. 

The treatment with C75 displayed only an OCR and MMP enhancement (Figure 1B), probably due to the increased availability of acetyl-CoA as fatty acid synthesis is inhibited. However, this molecule did not improve either the electron transport between Complexes I and III (Figure 1A) neither the ATP synthesis (Figure 1D), maintaining the uncoupling status like that observed in the untreated sample (Figure 1E). 

Since rapamycin is a negative modulator of aerobic metabolism [27,28], its effect on FA cells caused a further reduction of the Complexes I/III electron transport, the OCR, the MMP, and the ATP synthesis, without causing an improvement of the OxPhos efficiency (Figure 1). The opposite effect on OxPhos efficiency compared to that observed in the presence of quercetin could depend on the fact that only quercetin ameliorated the electron transport between Complexes I and III. 

**Figure 2:**
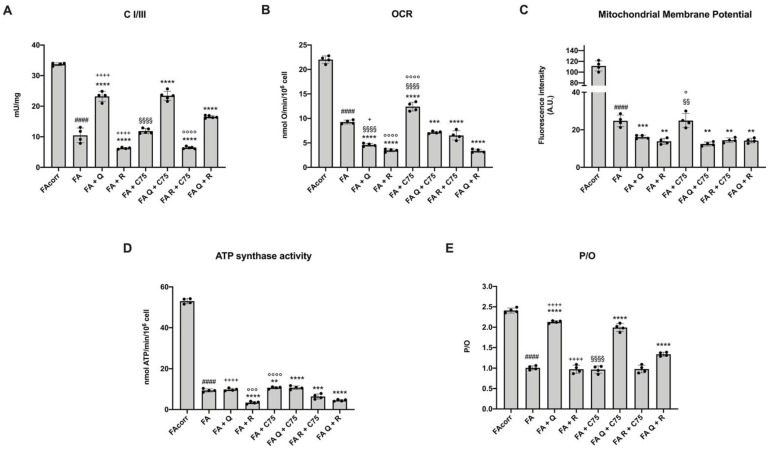
Modulation of aerobic metabolism in FA cells treated with quercetin, rapamycin, and C75, alone or in combination. All data represent experiments conducted after 48 h of drug treatments. (**A**) Electron transport between respiratory Complexes I and III. (**B**) Oxygen consumption rate (OCR). (**C**) Mitochondrial membrane potential measured by cytofluorimetric analysis. (**D**) Aerobic ATP synthesis through F_o_F_1_-ATP synthase. (**E**) P/O value, calculated as ratio between nmol of synthetized ATP (P) and consumed atomic oxygen (O). This parameter is a marker of OxPhos efficiency. (Q = quercetin alone; R = rapamycin alone; C75 = C75 alone; Q + C75 = quercetin/C75 combination; R + C75 = rapamycin/C75 combination; Q + R = quercetin/rapamycin combination). Each graph represents four independent experiments and data are expressed as mean ± S.D. For FA and FA-corr cells, data are representative of four different Fanc-A lymphoblast cell lines. Statistical analysis was performed via one-way ANOVA followed by Tukey multiple comparison test. #### indicates a *p* < 0.0001 between FA cells and FA-corr cell lines; **, ***, or **** indicate a *p* < 0.01, 0.001, or 0.0001, respectively, between untreated and treated FA cells. +, or ++++ indicate a *p* < 0.05, or 0.0001, respectively, between treatment with quercetin or rapamycin alone and quercetin/rapamycin combination; §§, or §§§§ indicate *p* < 0.01, or 0.0001 between treatment with quercetin or C75 alone and quercetin/C75 combination; °, °°°, or °°°° indicate a *p* < 0.05, 0.001, or 0.0001, respectively, between treatment with rapamycin or C75 alone and rapamycin/C75 combination.

For the same reasons, the quercetin combination with C75 maintained the positive effect associated with the flavonoid antioxidant action, whereas the combination with rapamycin partially reduced the improvement of OxPhos efficiency induced by quercetin. Conversely, the combination of C75 and rapamycin did not affect the P/O value, although it caused a further reduction of electron transport between Complexes I and III, the OCR, the MMP, and the ATP synthesis (Figure 2).

### 2.3. Quercetin, and Rapamycin and Their Combinations, but Not C75, Modulate the Anaerobic Glycolysis in FA Cells

**Figure 3:**
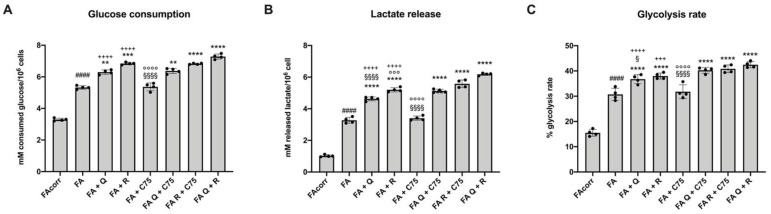
Changes in anaerobic glycolysis flux in FA cells treated with quercetin, rapamycin, and C75, alone or in combination. All data represent experiments conducted after 48 h of drug treatments. (**A**) Glucose consumption. (**B**) Lactate release in growth medium. (**C**) Glycolysis rate, evaluated as ratio between real lactate release and theoretical lactate release, which corresponds to twice concentration of glucose consumed. (Q = quercetin alone; R = rapamycin alone; C75 = C75 alone; Q + C75 = quercetin/C75 combination; R + C75 = rapamycin/C75 combination; Q + R = quercetin/rapamycin combination). Each graph represents four independent experiments and data are expressed as mean ± S.D. For FA and FA-corr cells, data are representative of four different Fanc-A lymphoblast cell lines. Statistical analysis was performed via one-way ANOVA followed by Tukey multiple comparison test. #### indicates a *p* < 0.0001 between FA cells and FA-corr cell lines; **, ***, or **** indicate a *p* < 0.01, 0.001, or 0.0001, respectively, between untreated and treated FA cells. +++, or ++++ indicate a *p* < 0.001, or 0.0001, respectively, between treatment with quercetin or rapamycin alone and quercetin/rapamycin combination; §, or §§§§ indicate *p* < 0.05, or 0.0001 between treatment with quercetin or C75 alone and quercetin/C75 combination; °°°, or °°°° indicate a *p* < 0.001, or 0.0001, respectively, between treatment with rapamycin or C75 alone and rapamycin/C75 combination.

The altered or reduced mitochondrial aerobic metabolism is often associated with a metabolic switch forwards the anaerobic glucose catabolism [29]. Therefore, to evaluate whether the treatment related OxPhos reduction was associated with an increment of anaerobic glycolysis, glucose consumption, lactate release, and the consequent glycolysis rate were investigated. Data reported in Figure 3 show that quercetin and rapamycin induced an increment of all parameters described above. Conversely, the single treatment with C75 did not produce any effects on the glycolysis flux. Regarding the treatment combination, the association of C75 with quercetin or rapamycin did not change the result of the single treatment with flavonoid or mTOR inhibitor. By contrast, the quercetin/rapamycin combination caused a further glycolysis rate increment compared to the single treatment due to the enhancement of glucose consumption and the lactate release.

### 2.4. Quercetin, C75, Rapamycin and Their Combination Modulated the Lipid Content in FA Cells

FA cells are characterized by an acetyl-CoA excess, which causes lipid droplets accumulation, increasing the risk of lipoperoxidation and the relative oxidative stress production [22,30]. Therefore, acetyl-CoA content, 3-ketoacyl-ACP reductase activity (a marker of fatty acid synthesis—FAS), and lipid content were evaluated in the presence of quercetin, C75, and rapamycin.

**Figure 4:**
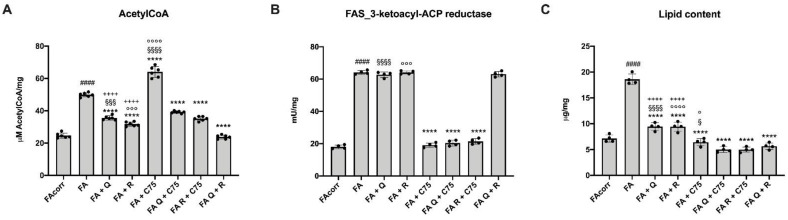
Modulation of fatty acid synthesis and lipid accumulation in FA cells treated with quercetin, rapamycin, and C75, alone or in combination. All data represent experiments conducted after 48 h of drug treatments. (**A**) Acetyl-CoA cellular accumulation. (**B**) 3-ketoacyl-ACP reductase activity, as marker of fatty acid synthesis (FAS). (**C**) Cellular lipid content. (Q = quercetin alone; R = rapamycin alone; C75 = C75 alone; Q + C75 = quercetin/C75 combination; R + C75 = rapamycin/C75 combination; Q + R = quercetin/rapamycin combination). Each graph represents four independent experiments and data are expressed as mean ± S.D. For FA and FA-corr cells, data are representative of four different Fanc-A lymphoblast cell lines. Statistical analysis was performed via one-way ANOVA followed by Tukey multiple comparison test. #### indicates a *p* < 0.0001 between FA cells and FA-corr cell lines; **** indicates a *p* < 0.0001 between untreated and treated FA cells. ++++ indicates a *p* < 0.0001 between treatment with quercetin or rapamycin alone and quercetin/rapamycin combination; §, §§§, or §§§§ indicate *p* < 0.05, 0.001, or 0.0001 between treatment with quercetin or C75 alone and quercetin/C75 combination; °, °°°, or °°°° indicate a *p* < 0.05, 0.001, or 0.0001, respectively, between treatment with rapamycin or C75 alone and rapamycin/C75 combination.

Quercetin and rapamycin caused a reduction in acetyl-CoA and lipid content (Figure 4A,C) but did not affect 3-ketoacyl-ACP reductase activity (Figure 4B). Thus, it is possible to speculate that their effect was due to OxPhos impairment and increased anaerobic glycolysis, leading to increased conversion of pyruvate to lactate instead of acetyl-CoA.

Treatment with C75, causing a reduction in the activity of 3-ketoacyl-ACP reductase (Figure 4B), resulted in lipid content reduction (Figure 4C). However, the FAS inhibition was associated with an accumulation of acetyl-CoA (Figure 4A).

Interestingly, the C75 combination with quercetin or rapamycin results in an additive effect, with the reduction of both acetyl-CoA and 3-ketoacyl-ACP reductase activity (Figure 4A,B), causing a further decrease in lipid content (Figure 4C). The quercetin/rapamycin combination also induced a sharp reduction of acetyl-CoA and lipid content than the individual treatments, even if they did not act on FAS activity (Figure 4B).

### 2.5. Quercetin, C75, Rapamycin, and Their Combination Decrease Oxidative Stress Production, the Relative Oxidative Damages, and Increase the Antioxidant Defenses in FA Cells

Dysfunctional aerobic metabolism is associated with an increment of oxidative stress production [31,32]. This appears true also in FA cells, which displayed an accumulation of reactive oxygen species and relative damages [10,11,12,15,16,17] caused by defective electron transport between Complexes I and III [13,26] and a low adaptative antioxidant response [19,26,33]. 

**Figure 5:**
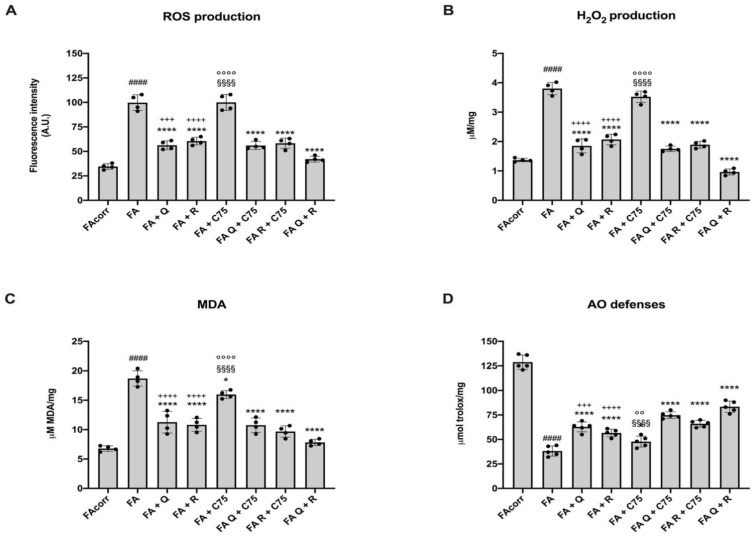
Modulation of oxidative stress production and antioxidant defenses in FA cells treated with quercetin, rapamycin, and C75, alone or in combination. All data represent experiments conducted after 48 h of drug treatments. (**A**) ROS production. (**B**) Hydrogen peroxide (H_2_O_2_) production. (**C**) Malondialdehyde content as a marker of lipid peroxidation. (**D**) Total cellular antioxidant defenses. (Q = quercetin alone; R = rapamycin alone; C75 = C75 alone; Q + C75 = quercetin/C75 combination; R + C75 = rapamycin/C75 combination; Q + R = quercetin/rapamycin combination). Each graph represents four independent experiments and data are expressed as mean ± S.D. For FA and FA-corr cells, data are representative of four different Fanc-A lymphoblast cell lines. Statistical analysis was performed via one-way ANOVA followed by Tukey multiple comparison test. #### indicates a *p* < 0.0001 between FA cells and FA-corr cell lines. **** indicates a *p* < 0.0001 between untreated and treated FA cells. +++, or ++++ indicate a *p* < 0.001, or 0.0001, respectively, between treatment with quercetin or rapamycin alone and quercetin/rapamycin combination; §§§§ indicates *p* < 0.0001 between treatment with quercetin or C75 alone and quercetin/C75 combination; °°, or °°°° indicate a *p* < 0.01, or 0.0001, respectively, between treatment with rapamycin or C75 alone and rapamycin/C75 combination.

The treatment with quercetin and rapamycin induced an evident decrease of ROS and hydrogen peroxide production, and the MDA accumulation, due to the OxPhos reduction (Figure 5A–C). In addition, both drugs induced a slight but significant increment of antioxidant (AO) defenses (Figure 5D). Conversely, C75 did not affect oxidative stress production, although it reduced the lipid peroxidation and increased the AO ability in FA cells, probably associated with the lipid accumulation decrement. 

Regarding the combinations, a further general positive effect on oxidative stress production and antioxidant defenses was observed only with the quercetin and rapamycin combination (Figure 5A–D). However, the combination of C75 with quercetin or rapamycin caused a more evident MDA reduction and the AO defenses improvement with respect to that observed with the C75 treatment alone (Figure 5C,D). 

### 2.6. The Treatment with Quercetin and C75, but Not Rapamycin, Improve the Energy Status in FA Cells

**Figure 6:**
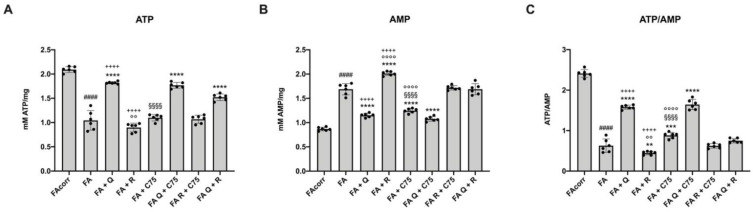
Modulation of ATP and AMP intracellular concentrations and consequent energy status in FA cells treated with quercetin, rapamycin, and C75, alone or in combination. All data represent experiments conducted after 48 h of drug treatments. (**A**) Intracellular ATP content. (**B**) Intracellular AMP content. (**C**) ATP/AMP ratio, as a marker of cellular energy status. (Q = quercetin alone; R = rapamycin alone; C75 = C75 alone; Q + C75 = quercetin/C75 combination; R + C75 = rapamycin/C75 combination; Q + R = quercetin/rapamycin combination). Each graph represents four independent experiments and data are expressed as mean ± S.D. For FA and FA-corr cells, data are representative of four different Fanc-A lymphoblast cell lines. Statistical analysis was performed via one-way ANOVA followed by Tukey multiple comparison test. #### indicates a *p* < 0.0001 between FA cells and FA-corr cell lines. **, ***, or **** indicate a *p* < 0.01, 0.001, or 0.0001 between untreated and treated FA cells. ++++ indicates a *p* < 0.0001 between treatment with quercetin or rapamycin alone and quercetin/rapamycin combination; §§§§ indicates *p* < 0.0001 between treatment with quercetin or C75 alone and quercetin/C75 combination; °°, or °°°° indicate a *p* < 0.01, or 0.0001, respectively, between treatment with rapamycin or C75 alone and rapamycin/C75 combination.

The OxPhos metabolism impairment and the need to counteract an excessive oxidative stress production induce a decrement in the energy status in FA cells compared to that of the control, as shown by the ATP/AMP ratio. This condition seems to be partially recovered by quercetin and C75 (Figure 6C). In detail, quercetin acted both on ATP and AMP levels, incrementing the first and reducing the last, probably thank to the improvement of electron transport and OxPhos efficiency. C75 only caused an AMP reduction due to the FAS inhibition (Figure 6A,B). Conversely, FA cells treated with rapamycin showed a lower ATP/AMP ratio compared to the untreated FA cells caused by the reduction of ATP level and the increment of AMP concentration (Figure 6). These opposite effects were detectable also with the two-by-two combinations of the three molecules (Figure 6). The quercetin and C75 combination confirmed the ATP/AMP ratio improvement observed with quercetin alone. Conversely, the combination with rapamycin and quercetin or C75 caused only a slight increment in the energy status with respect to the untreated sample but lower compared to that obtained with quercetin or C75 alone.

### 2.7. All Treatments Reduce the Damages and DNA Double-Strand Breaks Induced by Hydroxy Urea

**Figure 7:**
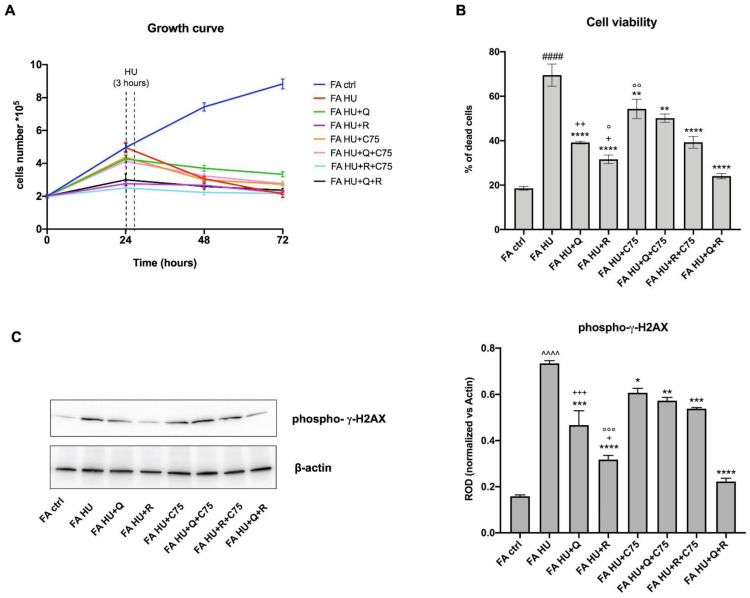
Effect of quercetin, rapamycin, C75, and their combination on damages and DNA double-strand breaks induced by hydroxyurea. Data reported in Panels B and C represent experiments conducted after 48 h of drug treatments. (**A**) Cell growth curves (**B**) Percentage of dead cells. (**C**) Left: WB signals against phospho-γ-H2AX and β actin, used as a housekeeping protein. Right: densitometric analysis of WB signal. (HU = hydroxyurea; Q = quercetin alone; R = rapamycin alone; C75 = C75 alone; Q + C75 = quercetin/C75 combination; R + C75 = rapamycin/C75 combination; Q + R = quercetin/rapamycin combination). Each graph represents four independent experiments and data are expressed as mean ± S.D. For FA and FA-corr cells, data are representative of four different Fanc-A lymphoblast cell lines. Statistical analysis was performed via one-way ANOVA followed by Tukey multiple comparison test. #### or ^^^^ indicates a *p* < 0.0001 between untreated FA (FA ctrl) cells and FA cells treated with HU (FA HU). *, **, ***, or **** indicate a *p* < 0.05, 0.01, 0.001, or 0.0001 between untreated and treated FA HU cells. +, ++, or +++ indicate a *p* < 0.05, 0.01, or 0.001, respectively, between treatment with quercetin or rapamycin alone and quercetin/rapamycin combination; °, °°, or °°° indicate a *p* < 0.05, 0.01, 0.001 between treatment with rapamycin or C75 alone and rapamycin/C75 combination.

To evaluate whether quercetin, rapamycin, C75, and their combinations are able to reduce the damages and DNA double-strand breaks induced by hydroxyurea (HU), cells were preincubated 24 h with the drugs, treated for 3 h with HU, and maintained in culture for the next 48 h. Results show that all treatments reduce the slope of HU-induced decrement of the FA cells growth, although without reversing it completely (Figure 7A). Also, the cellular death appears to be reduced. Specifically, quercetin, rapamycin, and their combination exert the maximum protective effect, while C75 seems to be less effective, although it is still able to significantly reduce the percentage of dead cells (Figure 7B).

In addition, the expression of phosphorylated γ-H2AX, a marker of DNA double-strand breaks, was evaluated by western blot analysis. Data show a trend similar to the reduction of cell death (Figure 7C). Specifically, the quercetin and rapamycin combination is the most effective treatment in the DNA breaks reduction, although both molecules used alone show already a remarkable protective role on DNA. Conversely, partial inhibition of the rapamycin protective effects appears when it is combined with C75. On the other hand, C75 exerts only a mild positive action, even if significant, compared to the sample treated with HU. 

## 3. Discussion

Since altered energy metabolism seems to play a pivotal role in FA pathogenesis, this work aims to understand if it is possible to correct the FA cells metabolic impairment, acting on one or more of the defective pathways.

As an antioxidant drug, we have chosen quercetin since this molecule acts as a ROS scavenger [34], modulating the OxPhos activity [35] and favoring the mitochondrial biogenesis [36,37]. In FA cells, quercetin treatment caused a simultaneous recovery of electron transfer between Complexes I and III and the OxPhos activity reduction. Since the slowed-down electron transport chain and the enhanced efficiency in the electron transport reduce the oxidative stress production [38], quercetin treatment also reduced the production of ROS, hydrogen peroxide, and consequent lipid peroxidation, causing a saving of energy and endogenous antioxidant defenses. Moreover, the reduction of oxidative stress production in the inner mitochondrial membrane, site of OxPhos metabolism, improved the coupling between oxygen consumption and ATP synthesis, enhancing the energy production efficiency, despite the slowdown of OxPhos activity. This induced an amelioration of the ATP/AMP ratio, which is also supported by an increment of the anaerobic glycolysis due to the OxPhos reduction. The quercetin effect on the lipid accumulation could depend on the anaerobic glycolysis acceleration and the consequent conversion of pyruvate to lactic acid instead of acetyl-CoA. On the other hand, quercetin regulates the lipid metabolism in several pathological models [39,40]. Interestingly, Li et al. have already observed that quercetin restores the IR cell signaling and ameliorates diabetes and obesity-prone phenotypes in FA mice, acting as a reducer of oxidative stress [41]. Quercetin also modulates lipid metabolism and IR by the expression of several lipid and glucose metabolism-related genes as fatty acid synthase and PPAR-γ [40] or glucose transporter type 4 (GLUT-4) and its translocation to the plasma membrane [42]. On the other hand, more in general, antioxidant molecules were used in vitro and in vivo to revert the altered lipid metabolism and IR [43,44,45,46]. 

mTOR seems to display several roles in cells affected by FA. Some authors suggest that mTOR modulates the DNA damage response since mTOR inhibition or deficiency causes suppressed expression of Fanc-D2 protein by phosphorylation and nuclear import of NF-kB [47,48,49]. In addition, in the stalled replication fork, mTOR interacts with Fanc-D2 stabilizing the replication site and protecting the new DNA strand from cellular exonucleases [47]. However, this effect seems linked to replication block alone but not on DNA interstrand crosslink. On the other hand, mTOR acts as a metabolic sensor for nutrients, energy, and stress [50], and it is hyperphosphorylated in FA cells [51]. For these reasons, FA cells were treated with rapamycin, the specific mTOR inhibitor. Data show that the drug enhanced the switch from aerobic to anaerobic, constitutively present in FA cells [13,52], causing a reduction of the oxidative stress and decreasing the acetyl-CoA concentration and the lipid droplets accumulation. However, rapamycin does not improve either the electron transport between complexes I and III or the efficiency of OxPhos. Therefore, no improvement in ATP/AMP ratio was shown despite the increase in anaerobic glycolysis. On the other hand, rapamycin inhibits the mitochondrial metabolism of T cells in vivo [53]. However, despite its weak effect on the FA energy metabolism amelioration, rapamycin treatment appeared highly effective in in vitro and in vivo aplastic anemia models [53,54,55], suggesting a role beyond metabolic regulation. Rapamycin preserves the hematopoietic stem and progenitor cells, reducing the inflammatory cytokines and enhancing the interleukin-10 level [55]. Moreover, rapamycin is considered a protector against oxidative stress in FA cells [56,57], and the slowdown of the cell cycle and proliferation could represent a gain of time to try to repair DNA damage [58]. 

The treatment with the FAS inhibitor C75 reduced the lipid content and the consequent lipid droplets formation, inducing a reduction of oxidative stress production. In fact, the presence of many lipids, easier the trigger of the lipoperoxidation that leads to the formation of aldehydes, including MDA, which can promote DNA interstrand crosslinks [59]. However, FAS-inhibition causes the acetyl-CoA accumulation, despite a weak but significant increment of OxPhos metabolism, probably because C75 did not correct the electron transport impairment. This aerobic metabolism enhancement associated with the FAS reduction could explain the slight increment in the ATP/AMP ratio due to the AMP concentration decrement. However, the reduction of lipid synthesis could play a pivotal role as a FA therapy. Specifically, 70% of FA patients display endocrine abnormalities, including dyslipidemia and metabolic syndrome, associated with cellular lipid droplets accumulation [24]. Moreover, the defective lipid metabolism could affect the hematopoietic stem cell differentiation since bone marrow failure is associated with a 3-fold increment of PPAR-γ a regulator of the adipocyte differentiation and lipid synthesis [60]. Moreover, the association between altered lipid metabolism and FA also emerged in a global FA miRNoma study, which shows the downregulation of miR-122 and miR-206 that regulate the cholesterol and fatty acid metabolism as well as the insulin signaling [61]

Regarding the combinations, the most evident improvement was observed with the quercetin/C75 mixture, which exerts an additive effect because of the first acts on the OxPhos defect and oxidative stress, and the second reduces lipid accumulation. Quercetin/C75 simultaneous action resulted, therefore, in a further improvement of both oxidative and energetic status. Conversely, the combination between rapamycin and C75 did not show a recovery of the energy status, despite the reduction of oxidative stress and lipid accumulation; while the rapamycin/quercetin combination results less effective than quercetin alone in improving electron transport and OxPhos efficiency, although a reduction in lipid content is observed.

All the modulating effects on the energy metabolism exerted by quercetin, rapamycin, C75, and their combination may depend on the slowdown of the cellular growth since C75 acts as a reducer of phospholipid availability due to the inhibition of fatty acid synthesis, and quercetin and rapamycin cause the decrement of mitochondrial metabolism and relative signaling. Moreover, the slowing down of cell growth can help activate DNA control and repair mechanisms for a longer time, even if they are defective. This hypothesis is confirmed by the cellular death reduction. In addition, all proposed drugs can reduce the DNA double-strand breaks and relative damages induced by HU, as shown by the decrease in the expression of phosphorylated-γ-H2AX.

## 4. Materials and Methods

### 4.1. Cell Lines and Treatments

Four different Fanc-A lymphoblast cell lines derived from four patients carried out different mutations of Fanc-A gene were obtained from the ‘‘Cell Line and DNA Biobank from Patients affected by Genetic Diseases’’ (G. Gaslini Institute)—Telethon Genetic Biobank Network (Project No. GTB07001) [62]. In addition, isogenic FA-corr cell lines, generated by Fanc-A Lymphoblast cell lines corrected with S11FAIN [63] retrovirus, were employed as control to maintain the characteristics of the FA cell lines except the Fanc-A gene mutation. All the cell lines were grown at 37 °C in RPMI supplemented with 10% fetal calf serum, glutamine, and antibiotics.

The cells were treated for 48 h with 10 µM quercetin, a flavonoid compound (Merck, cod: Q4951), 5 µM C75, a fatty acid synthesis inhibitor (Merck, cod: C5490), and 10 nM rapamycin, the mTOR inhibitor (Merck, cod: 37094). 

For the experiments in the presence of hydroxyurea (HU), cells were treated for 24 h with quercetin, C75, and rapamycin, as previously described. Afterward, 2 mM HU was added to the culture medium for 3 h. Then, cells were washed with a new growth medium containing the protective drugs and maintained in culture for 48 h.

To evaluate the cellular grow rate, FA cell lines were counted by Burker chamber after trypan blue staining.

### 4.2. Flow-Cytometric Assays for Cell Viability

Cellular viability was assessed by propidium iodide (PI) exclusion assays. Cells were incubated with 1 μg/mL PI (Sigma Chemical Co., St Louis, MO, USA) (5 min) and PI fluorescence measured by flow cytometry (FACSCalibur, Becton Dickinson, San Jose, CA, USA). Cells positive for PI fluorescence were considered dead cells [64].

### 4.3. Assay of the Electron Transport between Complexes I and III

The electron transfer from Complex I to Complex III was assayed spectrophotometrically, following the reduction of cytochrome c at 550 nm [26]. The reaction was started with the addition of 0.7 mM NADH. If the electron transport between Complex I and Complex III is conserved, the electrons pass from NADH to Complex I, then to Complex III via coenzyme Q, and finally to cytochrome c. 

### 4.4. Oxygen Consumption Rate Assay 

Oxygen consumption rate (OCR) was measured at 25 °C in a closed chamber, using an amperometric electrode (Unisense Microrespiration, Unisense A/S). For each assay, 200,000 cells permeabilized with 0.03 mg/mL digitonin for 1 min were employed. After the centrifugation at 1000× *g* to discard the digitonin excess, cells were resuspended in phosphate buffer saline (PBS). To induce the OCR, 10 mM pyruvate, 5 mM malate, and 0.1 mM ADP were added to stimulate the pathway composed by Complexes I, III, and IV. Data were expressed as nmol O/min/10^6^ cells [26].

### 4.5. Mitochondrial Trans Membrane Potential by Flow Cytometry 

Fresh cells were washed once with RPMI medium, incubated with 200 nM tetramethylrhodamine methyl ester (TMRM) (Invitrogen, Milan, Italy) for 10 min at 37 °C and immediately measured on a FacsCalibur flow cytometer (Becton Dickinson, San José, CA, USA). To exclude the unspecific staining, the same experiments were conducted in the presence of 50 nM of Carbonyl cyanide-4-(trifluoromethoxy)phenylhydrazone (FCCP), an uncoupling molecule. The analysis was confined to viable cells only, after gating procedures based on forward- and side-scatter features. Ten thousand cells per sample were analyzed [26].

### 4.6. Bioluminescent Luciferase F_o_-F_1_ ATP Synthase Assay

To evaluate the ATP synthesis through the FoF1-ATP synthase activity, 200,000 cells were incubated for 10 min at 37 °C in a medium containing: 10 mM Tris-HCl, pH 7.4, 100 mM KCl, 5 mM KH_2_PO_4_, 1 mM EGTA, 2.5 mM EDTA, and 5 mM MgCl_2_, 0.6 mM ouabain and 25 mg/mL ampicillin. Afterwards, ATP synthesis was induced by the addition of 10 mM pyruvate, 5 mM malate, and 0.1 mM ADP. The reaction was monitored for two minutes, every 30 s, in a luminometer (GloMax^®^ 20/20n Luminometer, Promega Italia, Milano, Italy), by the luciferin/luciferase chemiluminescent method, with ATP standard solutions between 10^−8^ and 10^−5^ M (luciferin/luciferase ATP bioluminescence assay kit CLSII, Roche, Basel, Switzerland). Data were expressed as nmol ATP produced/min/10^6^ cells [26].

The oxidative phosphorylation efficiency (P/O ratio) was calculated as the ratio between the concentration of the produced ATP and the amount of consumed oxygen in the presence of respiring substrate and ADP. In coupled conditions, this value is around 2.5 or 1.5 in the presence of pyruvate + malate or succinate, respectively [65]. Conversely, in the uncoupled status, this value decreases proportionally to the grade of the OxPhos inefficiency. 

### 4.7. Glucose Consumption and Lactate Release Assay

Glucose consumption was evaluated in the growth medium, following the reduction of NADP at 340 nm. The assay medium contained: 50 mM Tris-HCl pH 8.0, 1 mM NADP, 10 mM MgCl_2_, and 2 mM ATP in 1 mL final volume. Samples were analyzed spectrophotometrically before and after the addition of 4 µg of purified hexokinase plus glucose-6-phosphate dehydrogenase [66]. 

Lactate concentration in the growth medium was assayed spectrophotometrically, following the reduction of NAD^+^, at 340 nm. The assay medium contained: 100 mM Tris-HCl pH 8, 5 mM NAD^+^, 1 IU/mL of lactate dehydrogenase. Samples were analyzed spectrophotometrically before and after the addition of 4 µg of purified lactate dehydrogenase. Both data were normalized on the cells number [66]. 

The glycolysis rate was calculated as the percentage of real released lactate on the theoretical lactate production, which corresponds to twice the concentration of glucose consumed (as in an exclusive anaerobic metabolism, one glucose molecule is converted into two lactate molecules).

### 4.8. Evaluation of ATP/AMP Ratio 

ATP and AMP quantification was based on the enzyme coupling method [26]. To block all enzymatic activities 2.5% perchloric acid was added to 20 µg of total protein. The sample was centrifuged, and the supernatant was neutralized with 0.2 M K_2_CO_3,_ and used for both assays.

Briefly, ATP was assayed, following NADP reduction at 340 nm. The medium contained 50 mM Tris-HCl pH 8.0, 1 mM NADP, 10 mM MgCl_2_, and 5 mM glucose in 1 mL final volume. Samples were analyzed spectrophotometrically before and after the addition of 4 µg of purified hexokinase plus glucose-6-phosphate dehydrogenase.

AMP was assayed following the NADH oxidation at 340 nm. The medium contained 50 µg of cells homogenate, 100 mM Tris-HCl pH 8.0, 75 mM KCl, 5 mM MgCl_2_, 0.2 mM ATP, 0.5 mM phosphoenolpyruvate, 0.2 mM NADH, 10 IU adenylate kinase, 25 IU pyruvate kinase, and 15 IU of lactate dehydrogenase.

### 4.9. Evaluation of Acetyl-CoA Concentration

To evaluate the AcetylCoA concentration, the PicoProbe Acetyl CoA Assay Kit (Abcam, cod: ab87546) was employed, following the manufacture’s instruction.

### 4.10. 3-ketoacyl-ACP Reductase Assay 

The activity of 3-hydroxyacyl-CoA dehydrogenase was assayed as a marker of fatty acids beta-oxidation metabolism. The assay was performed spectrophotometrically at 340 nm, following the NADH oxidation in the presence of acetoacetyl-CoA [67]. The reaction mixture contained: 100 mM sodium phosphate, pH 6.0, 0.2 mM NADH and 0.1 mM acetoacetyl-CoA. 

### 4.11. Evaluation of Lipid Content 

The lipid content was evaluated by the Sulfo–Phospho–Vanillin assay [30]. Briefly, samples were incubated with 95% sulfuric acid at 95 °C for 20 min, quickly cooled, and evaluated at 535 nm. Afterwards, a solution of 0.2 mg/mL vanillin in 17% aqueous phosphoric acid was added to the samples, incubated for 10 min in the dark, and reevaluated at 535 nm. A triglycerides mix was used to obtain a standard curve.

### 4.12. Oxidative Stress, Lipid Peroxidation and Antioxidant Defenses Evaluation

To evaluate the reactive oxygen species (ROS) production by cytofluorimeter, cells were stained for 20 min at 37 °C with 2′,7′-dichlorodihydrofluorescein diacetate (H2DCFDA) (Thermo Fisher Scientific, Waltham, MA, USA). H2DCFDA is non-fluorescent but, inside the cell, it is cleaved to 2′,7′-dichlorofluorescein (H2DCF), which, in the presence of oxidants, is finally converted to the fluorescent DCF. Samples were analyzed on a CyAn ADP cytometer (Beckman Coulter, Mountain View, CA, USA) equipped with a 15-mW 488-nm argon ion laser. The plot of all physical parameters (forward scatter (FSC) versus side scatter (SSC)) was used to set the gate that delimits debris and aggregates. Ten thousand cells per sample were analyzed [26].

H_2_O_2_ content was evaluated by the Fluorimetric Hydrogen Peroxide Assay Kit (Sigma–Aldrich, cod: MAK165), following the manufacturer’s instructions.

To assess the lipid peroxidation, the malondialdehyde (MDA) concentration was evaluated, using the thiobarbituric acid reactive substances (TBARS) assay [68]. This test is based on the reaction of MDA, a breakdown product of lipid peroxides, with thiobarbituric acid. The TBARS solution containing: 15% trichloroacetic acid in 0.25 N HCl and 26 mM thiobarbituric acid. To evaluate the basal concentration of MDA, 600 µL of TBARS solution was added to 50 µg of total protein dissolved in 300 µL of Milli-Q water. The mix was incubated for 40 min at 100 °C. After the sample was centrifuged at 14,000 rpm for 2 min and the supernatant was analyzed spectrophotometrically, at 532 nm.

The general antioxidant defenses and the relative level of scavengers were evaluated by the Total Antioxidant Capacity Assay Kit (Sigma–Aldrich, cod: MAK187), following the manufacturer’s instructions.

### 4.13. Western Blot Analysis

Denaturing electrophoresis (SDS-PAGE) was performed on 4–20% gradient gels (Bi- oRad) (Hercules, CA, USA), loading 30 μg of protein homogenate for each sample. After the electrophoresis separation, proteins were transferred onto the nitrocellulose membrane, which was blocked with 5% BSA. Membrane was incubated with a mouse antibody against phosphorylated-γ-H2AX (Merck Millipore, cod: 05-636), a marker of DNA double-strand breaks, and a mouse antibody against β-actin (Santa Cruz Biotechnology, cod: sc-1616), used as housekeping protein. All primary antibodies were diluted 1:10,000 in PBS plus 0.15% tween (PBSt). Bands were detected and analyzed for optical density using an enhanced chemiluminescence substrate (ECL, BioRad), a chemiluminescence system (Alliance 6.7 WL 20 M, UVITEC), and UV1D software (UVITEC). Bands of interest were normalized for actin levels in the same membrane. 

### 4.14. Statistical Analysis

Data were analyzed by one-way ANOVA followed by Tukey multiple comparison test, using Instat software (GraphPad Software, Inc., La Jolla, CA, USA). Data are expressed as mean ± standard deviation (S.D.) from 4 independent measurements performed in triplicate. In the figures, S.D. is shown as error bars. An error probability with *p* < 0.05 was selected as significant.

## 5. Conclusions

Although the link between pathogenic variants of FA genes and altered energy metabolism was not yet described, these biochemical alterations appear to play a pivotal role in the disease, suggesting that FA should be considered a multifactorial disease. In fact, the mitochondrial metabolic defect causes a wired cellular unbalance, which leads to altered insulin sensitivity, dyslipidemia, and increment of oxidative stress and the consequent DNA damage. At a first analysis, the slowdown of mitochondrial activity could solve, or at least reduce, some problems related to mitochondrial dysfunction. In other words, the silencing of mitochondrial activity could reduce the damage associated with the metabolic switch due to passage from the bone marrow niche to the bloodstream [26]. However, the mitochondrion plays more than just an energetic role in bone marrow, as it acts as a regulator of the hematopoietic stem cells [69]. Thus, the work aims to reduce the stress induced by impaired mitochondrial functionality without completely losing its activity, which is a critical signal for stem pool maintenance. In other words, the multidrug approach may represent a proper balance to modulate mitochondrial activity while reducing cellular stress. Specifically, from a metabolic point of view, the quercetin and C75 combination seems to be the most effective in achieving this new balance, as it acts on two different targets (OxPhos and lipid metabolism), both aimed at reducing oxidative stress. Regarding the rescue of DNA damages, the quercetin and rapamycin combination appears to be the most efficient, as they induce the cell cycle slowdown, favoring the DNA control and recovery. However, this study represents the first attempt at a multifactorial approach to FA, and further investigation will be necessary to translate these in vitro results into an in vivo application.

## Data Availability

The data presented in this study are available on request from the corresponding author. The data are not publicly available due to are part of a broad database that also collects personal patient’ data.
